# The Proper Use of Antipyrine

**Published:** 1886-11

**Authors:** 


### *THE PROPER USE OF ANTIPYRINE.*

Pavay has employed this drug in a large number of cases, and gives some useful rules for its administration. He adopts a middle course in regard to dosage. When the temperature does not exceed 103° F., he divides 31 grains into three powders and administers one powder every half hour. If the thermometer registers 104°, three doses are given, as before, each dose consisting of 15½ grains. With a temperature of 105° and above, he gives 62 grains in four doses, half an hour apart. It is seldom, the writer asserts, that the temperature fails to fall from 2–4°, and to remain lowered from six to sixteen hours. If for any reason the stomach will not retain the drug, it may be given by the rectum in doses of from 30–45 grains, or hypodermically in a fifty per cent. solution.—*New York Med. Jour.* Sept. 1886.

